# Influence of nutrient intake on antioxidant capacity, muscle damage and white blood cell count in female soccer players

**DOI:** 10.1186/1550-2783-9-32

**Published:** 2012-07-19

**Authors:** Leyre Gravina, Fatima Ruiz, Elena Diaz, Jose Antonio Lekue, Aduna Badiola, Jon Irazusta, Susana Maria Gil

**Affiliations:** 1 Department of Physical Education and Sport, Faculty of Physical Activity and Sports Science, University of the Basque Country (UPV/EHU), Portal de Lasarte 71, 01007, Gasteiz, Spain; 2 Department of Nursing I, School of Nursing, University of the Basque Country (UPV/EHU), B° Sarriena s/n, 48940, Leioa, Bizkaia, Spain; 3 Department of Physiology, Faculty of Medicine and Dentistry, University of the Basque Country (UPV/EHU), B° Sarriena s/n, 48940, Leioa, Bizkaia, Spain; 4 Medical Services, Athletic Club de Bilbao, Basque Country, Garaioltza, 147-48196, Lezama, Bizkaia, Spain

**Keywords:** Immune response, Inflammation, Antioxidant enzymes, Dietary intake, Cell breakdown, Oxidative stress, Female athletes, Macronutrients, Micronutrients, Football

## Abstract

**Background:**

Soccer is a form of exercise that induces inflammatory response, as well as an increase in free radicals potentially leading to muscle injury. Balanced nutritional intake provides important antioxidant vitamins, including vitamins A, C and E, which may assist in preventing exercise-related muscle damage. The purpose of the present study was to determine the effect of macro/micronutrient intake on markers of oxidative stress, muscle damage, inflammatory and immune response in female soccer players.

**Methods:**

Twenty-eight female players belonging to two soccer teams of the same professional soccer club participated in this study after being informed about the aims and procedures and after delivering written consent. Each team completed an 8-day dietary record and played one competition match the same week. Participants were divided into two groups: the REC group (who complied with recommended intakes) and the NO-REC group (who were not compliant). Laboratory blood tests were carried out to determine hematological, electrolytic and hormonal variables, as well as to monitor markers of cell damage and oxidative stress. Blood samples were obtained 24 h before, immediately after and 18 h after official soccer matches. Student *t*-test or Mann–Whitney *U*-test was used to compare both groups throughout the match.

**Results:**

At rest, we observed that the REC group had higher levels of total antioxidant status (TAS), glutathione peroxidase (GPx), and lower levels of creatine kinase (CK) and lactate dehydrogenase (LDH) in comparison to the NO-REC group. Immediately after the match, levels of TAS, GPx, superoxide dismutase (SOD), LDH and % lymphocytes were higher and the % of neutrophils were lower in the REC group compared to the NO-REC group. These differences were also maintained 18 h post-match, only for TAS and GPx.

**Conclusions:**

Our data reveal an association between nutritional intake and muscle damage, oxidative stress, immunity and inflammation markers. The benefit of the intake of specific nutrients may contribute to preventing the undesirable physiological effects provoked by soccer matches.

## Background

Many studies have examined the physiological alterations that occur in the body following a soccer match. These effects depend on the exercise intensity of the match and the playing position of each player. In fact, this physical exercise has been considered by some as a muscle-damaging exercise [1] due to the important alterations in some biochemical parameters which are surrogate markers of skeletal muscle damage or injury.

Skeletal muscle damage is characterized by delayed-onset muscle soreness, muscle fiber disarrangement, muscle protein release into plasma, acute-phase immune response, and a decrease in performance [2]. Moreover, exercise-induced muscle damage is associated with increased production of reactive oxygen species (ROS) and other inflammatory molecules [3]. Under normal physiological conditions, the cellular antioxidant system removes these deleterious molecules. However, oxidative stress occurs when there is an imbalance between the production of free radicals and antioxidant defense. Oxidative stress may be involved in the aging process, cell damage, various pathologies, muscular fatigue, and overtraining (specifically inadequate recovery) [4].

Exercise has also been shown to alter total blood cell counts and the lymphocyte proliferative response [5], as well as it could cause neutrophilia (an elevated circulating neutrophil count), in male [6] and female [7] soccer players. Not only does soccer appear to induce a marked inflammatory response during recovery [8], but it also increases the concentration of antioxidant molecules, such as total antioxidant status (TAS), glutathione peroxidase (GPx), thiobarbituric acid reactive substances (TBARS), protein carbonyls (PC) and uric acid [9].

Currently, the vast majority of published studies have compared the oxidant and antioxidant status of soccer players and sedentary controls. These studies have revealed that TAS, superoxide dismutase (SOD), uric acid, ascorbic acid and tocopherol plasma levels are all higher in soccer players than in sedentary controls, while malondialdehyde (MDA) levels were lower [10,11]. These findings suggest that exercise training increases the production of free radicals and as a result, the utilization of natural endogenous antioxidants. Therefore, appropriate nutrition is likely to be vitally important in maintaining adequate antioxidant defense mechanisms.

It is widely accepted that the practice of healthy, balanced nutrition is beneficial for enhanced athletic performance [12]. To date, numerous studies have attempted to reveal the cost and benefits of the intake of nutritional supplements in athletic performance. However, most of the studies are controversial and further research is needed in order to arrive at a better understanding of how nutrition influences performance. Supplements studied thus far in humans include zinc, dietary fat, plant sterols, antioxidants, glutamine and carbohydrate. On the one hand, some authors have found that none of these supplements are an effective countermeasure to exercise-induced immune suppression except for carbohydrate beverages [13-15]. On the other hand, it has been suggested that the consumption of antioxidant supplements improves the antioxidant defense system, leading to reduced exercise-induced oxidative stress [16,17]. While some studies have provided scientifically-based nutritional guidelines for soccer players, few have examined the influence of nutrition on the physiological consequences of playing a soccer match. The aim of this study was thus to determine the effect of macro/micronutrient intake on the antioxidant response, cell damage, and on the inflammatory and immunity responses induced by a physical stresses of the soccer match in high level female soccer players.

## Methods

### Subjects

Two female soccer teams (*n* = 28) participated in this study after being informed about the aims, experimental protocol and procedures, and providing informed consent. They played in two categories, one in the First Division and the other team in the Second Division of the Spanish League. Players were 21 ± 6 years old, weighed 61 ± 8.4 kg and their body fat and muscle percentages were 16.7 ± 3.2% and 47 ± 2.6%, respectively (means ± S.D). All players engaged in two hours of training per training day and played one match every weekend. Also it is interesting to note that they played for a professional soccer club and they had been one of the best teams in their category during the last years. Precisely, the team from 1st Division had won the championship consecutively for 5 years. The experimental protocol was approved by the Ethical Committee of Cruces Hospital (Bizkaia).

### Dietary intake

Players registered their food and drink intake for 8 days including a match day. They were provided with a scale (Soehnle 1245) and a special booklet, designed for the purpose of this research. All participants were fully instructed on how to weigh and how to record all their food and beverages to be consumed. Players weighed food and drinks before eating/drinking, and at the end of the meal they again weighed the left- overs, to calculate net intake. If they had a meal with a dish made of a mixture of food (e.g. vegetables, rice, meat etc.), they were asked to record them all. The ingredients of the meals were thoroughly registered for both quantity and quality characteristics; for example type of oil, type of bread/milk etc. They were requested to follow their customary eating patterns during the recording days. On completion of the 8-day recording period, participants were asked to return their completed diary for analysis. If players were taking supplements, these were included in the analysis. Food records were reviewed for completeness and missing details were clarified with the player. The dietary records were introduced into a database by the first author and supervised by a physician-nutritionist. All the dietary records were analyzed using the nutrient analysis software DIAL V.1 [18]. This analysis provided detailed information about the intake of calories, macronutrients, vitamins and minerals.

### Experimental procedures and blood sampling

Anthropometric measurements and laboratory blood tests were carried out on the participants. Blood samples were obtained 24 h before, immediately after and 18 h after official soccer matches. In order to obtain representative values, data were collected from four matches, two in February and two in April (one match for each team), which were in the middle and at the end of the season, respectively.

Blood samples were drawn from the antecubital vein with participants in the seated position. Three ml of blood were collected into two vacutainer tubes containing EDTA for leukocyte analysis and antioxidant enzyme determination, and 7 ml in vacutainer tubes containing gelose for biochemical analysis. For antioxidant enzyme analysis, blood samples were centrifuged and the serum was stored at −80 °C until analysis.

Blood parameters were determined by standard clinical laboratory techniques. Those related to hematimetry and white blood cells were measured using an automated hematology analyzer (Sysmex XE-2100, Roche, Japan). Vitamin B12, folic acid and hormones were measured using a Modular Analytics SWA (Roche, Germany/Japan) analyzer. Other biochemical parameters (glucose, uric acid, triglyceride, cholesterol, cholesterol, high density lipoprotein (HDL), low density lipoprotein (LDL), glutamine- oxaloacetic transaminase (GOT), glutamine-pyruvic acid-transaminase (GPT), gamma- glutamyl transpeptidase (GGT), alkaline phosphatase (FA), total and direct bilirubin, lactate dehydrogenase (LDH), total creatine kinase (CK), sodium, potassium, chlorine, bicarbonate, calcium, phosphorus, magnesium, total proteins, albumin, urea, creatinine, C-reactive protein (CRP), iron, transferrin, and ferritin were measured using a CN- Cobas Integra 400 Plus (Roche, Germany/Japan).

Enzymatic determinations of the plasma concentration of total antioxidant status (TAS), superoxide dismutase (SOD), glutathione reductase (GR) and glutathione peroxidase (GPx) were performed in our laboratory. TAS was determined using the NX2332 kit from Randox laboratories. GR was measured photometrically using the RS 2368 kit.

(Randox), SOD using the Superoxide Dismutase Assay Kit (Cayman chemical) and GPx with the Glutathione Peroxidase Assay kit (Cayman chemical).

### Statistical analysis

Means and standard deviations (SD) were calculated for all measured variables. Dietary Reference Intakes (DRI) [19-22] were considered to be reference values of adequate intake. After pooling all participants together for each nutrient, participants were classified into two groups: those players who met the recommendation according to the DRI (REC group) and those who did not meet recommendations (NO-REC group). As two soccer matches were considered, these data from 56 measurements at each time point in the match were included in the statistical analysis. Both groups (REC and NO-REC groups for each nutrient) were checked for normality using the Shapiro-Wilk (if n < 50) and the Kolmogorov-Smirnov (if n > 50) statistical tests. Depending on the normality of these data, the Student-*t* test (parametric data) or the Mann–Whitney *U*-test (non-parametric data) test was used. Thus, we compared the different blood parameters at each of the three time points during the soccer match (before, immediately after and 18 h after) for the REC and NO-REC groups. The Statistical Package for the Social Sciences (SPSS Inc., version 16.0) was used for all analyses. The significance level was set at *p* ≤ 0.05.

## Results

### General considerations in nutritional profile

The mean of reported energy (n = 42) calculated from 8-d records was 2271 ± 578 kcal/day. The nutrition pattern of these players was characterized by a higher protein (15 ± 2%), a lower carbohydrate (44.3 ± 6%) and a higher fat diet (37 ± 7%), compared with the nutritional recommendations (protein 10-12%; carbohydrates 50-60%; fat <35%). Fiber ingestion was also lower (20 ± 7 g/day) than the recommendation (25–26 g/day). We also observed a higher cholesterol intake (340 mg/day) than recommended (<300 mg). Fatty acid intake was adequate, except for saturated fatty acids (12.4 ± 3% contribution to total energy). Regarding specific fatty acids, two ratios were also considered: polyunsaturated/saturated fatty acids (PUFAs/SFAs) and polyunsaturated + monounsaturated/saturated fatty acids (PUFAs + MUFAs/SFAs). Both ratios were also lower (0.4 ± 0.2 PUFAs/SFAs and 1.8 ± 0.4 PUFAs + MUFAs/SFAs) than the recommended values for PUFAs/SFAs (>0.5) and PUFAs + MUFAs/SFAs (>0.2). As regards vitamins and minerals, female players presented sub-optimal ingestion of folic acid (230 ± 100 μg/day), vitamin D (3.3 ± 2 μg/day), iodine (94.5 ± 30 μg/day), magnesium (315 ± 97 mg/day) and potassium (2973 ±971 mg/day). The rest of ingested micronutrients were found to comply with the Recommended Dietary Intakes (DRI).

### Nutritional intake vs. Blood parameters

Regarding the relationship between the intake of different nutrients and the blood parameters measured for the soccer matches, we only present those findings which were statistically significant.

**Figure 1:**
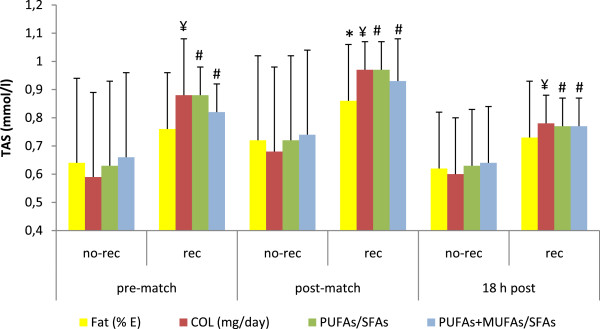
**Fat intake and total antioxidant status (TAS) in female players throughout a soccer match.** Differences between rec (compliance with recommendations) and no-rec groups: ¥ *p* < 0.001, #*p* < 0.01, * *p* < 0.05. Abbreviations: No-rec, players who did not comply with the recommended intake; Rec, players who complied with the recommendation intake; COL, cholesterol; PUFA, polyunsaturated fatty acid; SFA, saturated fatty acid; MUFA, monounsaturated fatty acid. Data are expressed as mean ± SD.

**Figure 2:**
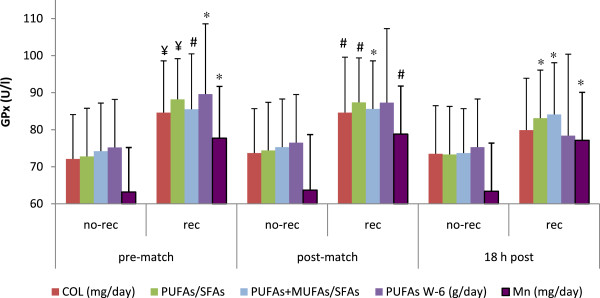
**Nutrient intake and glutathione peroxidase activity (GPx) in female players throughout a soccer match.** Differences between the rec and no-rec groups: ¥*p* < 0.001, # *p* < 0.01, * *p* < 0.05. Abbreviations: W6, W6 fatty acid; Mn, manganese; other abbreviations as in Figure 1. Data are expressed as mean ± SD.

**Figure 3:**
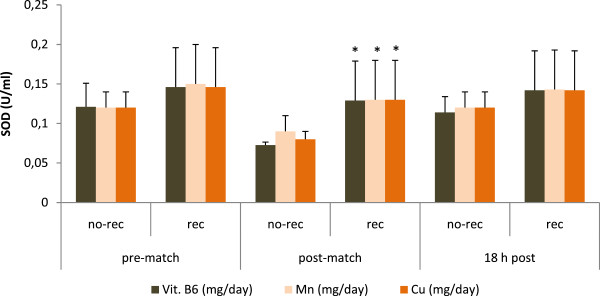
**Nutrient intake and superoxide dismutase activity (SOD) in female players throughout a soccer match.** Differences between rec and no-rec group: *p < 0.05. Abbreviations: B6, vitamin B6, Mn, manganese; Cu, copper; other abbreviations as in Figure 1. Data are expressed as mean ± SD.

**Figure 4:**
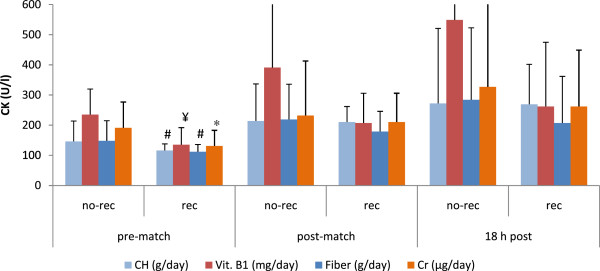
**Nutrient intake and creatine kinase (CK) activity in female players throughout a soccer match.** Differences between rec and no-rec group: ¥ *p* < 0.001, #*p* < 0.01, * *p* < 0.05. Abbreviations: CH, carbohydrate; Vit. B1, vitamin B1; Cr, chromium; other abbreviations as in Figure 1. Data are expressed as mean ± SD.

**Figure 5:**
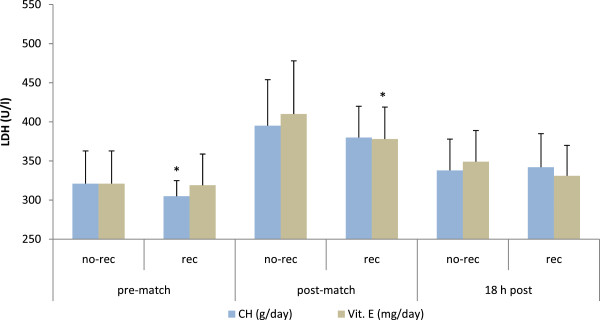
**Nutrient intake and lactate dehydrogenase activity (LDH) in female players throughout a soccer match.** Differences between rec and no-rec group: *p < 0.05. Abbreviations: CH, carbohydrate; Vit. E, vitamin E; other abbreviations as in Figure 1. Data are expressed as mean ± SD.

**Figure 6:**
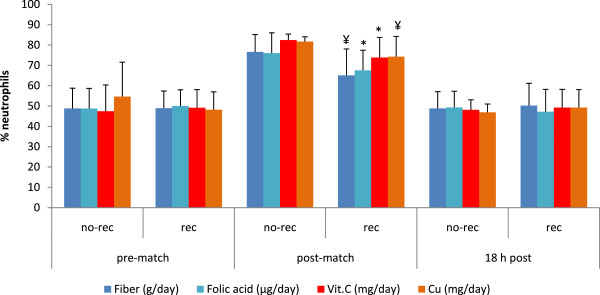
**Nutrient intake and neutrophil percentage in female players throughout a soccer match.** Differences between rec and no-rec group: ¥ *p* < 0.001, # *p* < 0.01, *p < 0.05. Abbreviations: Vit. C, vitamin C; Cu, copper; other abbreviations as in Figure 1. Data are expressed as mean ± SD.

**Figure 7:**
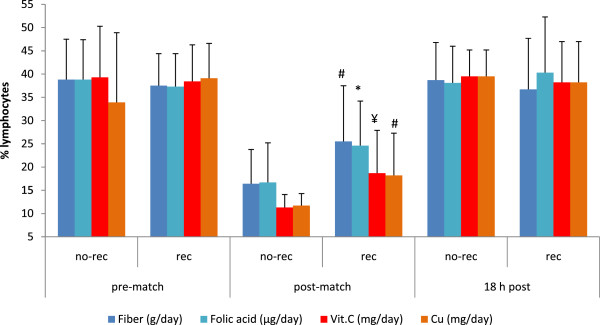
**Nutrient intake and lymphocyte percentage in female players throughout a soccer match.** Differences between rec and no-rec group: ¥ *p* < 0.001, # *p* < 0.01, *p < 0.05. Abbreviations: Vit. C, vitamin C; Cu, copper; other abbreviations as in Figure 1. Data are expressed as mean ± SD.

a) Influence of nutrition on oxidative markersResponses of oxidative markers are illustrated in Figure 1, 2 and 3. Figure 1 summarizes the influence of fat intake on antioxidant capacity measured before and after playing soccer matches. Those players whose fat intake was adequate (fat contribution to total energy ingested was lower than 35%) had higher levels of TAS immediately after matches (0.72 ± 0.3 vs. 0.86 ± 0.2mmol/l, *p* < 0.05). Also, immediately after the game, players with compliant cholesterol consumption (lower than 300 mg/day) showed higher levels of this antioxidant capacity (0.68 ± 0.3 vs. 0.97 ± 0.1mmol/l, *p* < 0.001). This difference was also maintained at rest (0.59 ± 0.3 vs. 0.88 ± 0.2mmol/l, *p* < 0.001) and 18 h post-match (0.60 ± 0.2 vs. 0.78 ± 0.1 mmol/l, *p* < 0.001). Moreover, players with compliant PUFAs/SFAs ratio (< 0.5) also exhibited a higher antioxidant capacity at rest (0.63 ± 0.3 vs. 0.88 ± 0.1 mmol/l, *p* < 0.01), immediately post-match (0.72 ± 0.3 vs. 0.97 ± 0.1 mmol/l, *p* < 0.01) and 18 h later (0.63 ± 0.2 vs. 0.77 ± 0.1 mmol/l, *p* < 0.01). Similar differences were also found for the PUFAs + MUFAs/SFAs ratio, with higher levels at rest (0.66 ± 0.3 vs. 0.82 ± 0.1 mmol/l, *p* < 0.01), immediately after a match (0.74 ± 0.3 vs. 0.93 ±0.2 mmol/l, *p* < 0.01) and 18 h post-match (0.64 ± 0.2 vs. 0.77 ± 0.1 mmol/l, *p* < 0.01). The influence of fat and manganese intake on GPx activity was also examined (Figure 2). Players presented lower levels of GPx activity at basal levels when they were not compliant for: cholesterol (72.1 ± 12 vs. 84.6 ± 14 U/l, *p* < 0.001), PUFAs/SFAs ratio (72.8 ± 13 vs. 88.2 ± 11 U/l, *p* < 0.001), PUFAs + MUFAs/SFAs ratio (74.2 ± 13 vs. 85.5 ± 15 U/l, *p* < 0.01), omega-6 fatty acids (75.2 ± 13 vs. 89.6 ± 19 U/l, *p* < 0.05) and manganese intake (63.2 ± 12 vs. 77.7 ± 14 U/l, *p* < 0.05). Similarly, GPx levels were lower immediately after the match for non-compliant consumers of: cholesterol (73.7 ± 12 vs. 84.6 ± 15 U/l, *p* < 0.01), PUFAs/SFAs ratio (74.4 ± 13 vs. 87.4 ± 12 U/l, *p* < 0.01), PUFAs + MUFAs/SFAs ratio (75.3 ± 13 vs. 85.6 ± 13 U/l, *p* < 0.05) and manganese (63.7 ± 15 vs. 78.8 ± 13 U/l, *p* < 0.01). After 18 hours post-match, the activity of GPx enzyme was lower for non-compliant consumers of PUFAs/SFAs ratio (73.3 ± 13 vs. 83.1 ± 13 U/l, *p* < 0.05), PUFAs + MUFAs/SFAs ratio (73.7 ± 12 vs. 84.1 ± 14 U/l, *p* < 0.05) and manganese (63.1 ± 13 vs. 77.1 ± 13 U/l, *p* < 0.05). The influence of vitamin B6, manganese and copper intake on the activity of superoxide dismutase enzyme (SOD) is illustrated in Figure 3. Players who complied with the recommendation for vitamin B6 (1.3 mg/day) presented higher SOD activity at the conclusion of the game (0.073 ± 0.004 vs. 0.129 ± 0.05 U/ml, *p* < 0.05). Moreover, the activity of SOD was lower when players did not meet with the recommendations for manganese (1.8 mg/day) (0.09 ± 0.02 vs. 0.13 ± 0.05 U/ml, *p* < 0.05) and copper (0.9 mg/day) (0.08 ± 0.01 vs. 0.13 ± 0.05 U/ml, *p* < 0.05) immediately after the match.

b) Influence of nutrition on cell damage markersExercise-induced cell damage is illustrated in Figure 4 and 5. Figure 4 shows the influence of carbohydrate, vitamin B1, fiber and chromium intake on creatine kinase activity measured before and after playing a soccer game. Creatine kinase activity was lower at basal levels in those players who were compliant in intakes of: carbohydrates (50-60% of total energy) (146 ± 68 vs. 116 ± 22 U/l, *p* < 0.01), vitamin B1 (1.1 mg/day) (235 ± 85 vs. 135 ± 57 U/l, *p* < 0.001), fiber (25 g/day) (148 ± 67 vs. 112 ± 24 U/l, *p* < 0.01) and chromium (25 μg/day) (191 ± 86 vs. 131 ± 52 U/l, *p* < 0.05). Figure 5 summarizes the influence of carbohydrate and vitamin E intake on the activity of lactate dehydrogenase (LDH). At basal levels, LDH activity was higher in those players who were not compliant for carbohydrate (321 ± 42 vs. 305 ± 20 U/l, *p* < 0.05) and for vitamin E intake (8 mg/day) immediately after the match (410 ± 68 vs. 379 ± 41 U/l, *p* < 0.05).

c) Influence of nutrition on white blood cellsImmune and inflammation responses are illustrated in Figure 6 and 7. Figure 6 shows the influence of fiber, folic acid, vitamin C and copper intake on the variation of percentage of neutrophils induced by a soccer match. Neutrophil percentages were lower immediately post-match in those players who were compliant for intakes of fiber (77 ± 8.6 vs. 65 ± 13%, *p* < 0.001), folic acid (76 ± 10 vs. 68 ± 10%, *p* < 0.05), vitamin C (82 ± 3 vs. 74 ± 10%, *p* < 0.05) and copper (82 ± 2.4 vs. 74 ± 10%, *p* < 0.001). Figure 7 represents the influence of all these nutrients on lymphocyte percentages associated with soccer matches. Higher percentages of lymphocytes immediately post-match were observed in players who were compliant in their intakes of fiber (16 ± 7.5 vs. 26 ± 12%, *p* < 0.01), folic acid (17 ± 8.5 vs. 25 ± 9.6%, *p* < 0.05), vitamin C (11 ± 2.6 vs. 19 ± 9.2%, *p* < 0.001) and copper (12 ± 2.6 vs. 18 ± 9.1%, *p* < 0.01).

Fig6, Fig7

## Discussion

Currently, there is a lack of information regarding the influence of nutrition on the performance and physiological response associated with playing soccer. The present research provides evidence that an appropriate nutritional intake improves the antioxidant capacity of soccer players and influences the activity of the principal antioxidant enzymes (such as superoxide dismutase and glutathione peroxidase) that protect against the potentially damaging effects of oxidative stress. Furthermore, some specific macronutrients and micronutrients diminish the negative physiological impact of playing a soccer match, since changes in some markers related to cell damage, inflammation and immunity were found.

Oxidative stress plays a vital role in a number of physiological processes, including contributing to a healthy immune system, since free radicals can function as signaling messengers for many cellular metabolic processes [23,24]. Venous blood samples can be analyzed for radical content to ascertain the degree of oxidative stress due to factors, such as exercise like soccer [6,8,10,25]. Recently, the responses of circulating levels of markers of oxidative stress and antioxidant status during recovery from a soccer game have been determined [9]. These authors found that thiobarbituric acid reactive substances (TBARS), C-protein reactive, uric acid, GPx and TAS concentrations were increased during recovery. Our study indicates that the levels of some of these protective markers could be enhanced if the fat intake of soccer players is controlled. We found that lower cholesterol intake, as well as a lower proportion of ingested saturated fatty acids, with respect to polyunsaturated + monounsaturated fatty acids, seems to provide better antioxidant capacity, since TAS and GPx activity were higher at baseline levels, before and after playing a soccer match. Other studies have found similar relationships in rats after having been fed with high-fat diets [26,27]. In keeping with our findings, a regular intake of optimized sunflower oils (oil enriched in monounsaturated fatty acids) has recently been reported to help improve lipid status and reduce lipid peroxidation in plasma [28]. As far as fat intake is concerned, we have also found that omega-6 fatty acids enhance glutathione peroxidase activity at basal levels of players who complied the recommendation intake. The beneficial effects of omega-3 and its relationship with antioxidant capacity have been amply demonstrated. However, our results also illustrate the beneficial influence of omega-6, which has been reported before [29].

Endogenous enzymes such as superoxide dismutase and glutathione peroxidase are components of the body’s primary defense system. They modulate the synthesis of cell signaling molecules which lead to the regulation of oxidative stress [30]. Dietary components such as the micronutrients manganese, zinc, copper and selenium can act as co-factors for endogenous enzymes. Superoxide dismutase, for example, has zinc, copper and manganese dependent forms. Thus, when there is a deficiency of these nutrients, the activity of the endogenous enzyme can be jeopardized [23]. Our study reveals a significant association between a higher dietary intake of manganese and copper and a higher activity of this enzyme, especially at the conclusion of the match. Several studies have demonstrated enhanced concentration of antioxidant enzymes after exercise. Most of these studies involved submaximal or maximal effort aerobic exercise [31] and high-intensity interval training [32,33]. These authors proposed that oxidative stress and the necessity to protect against oxidative damage may be responsible, at least partially for the elevation in the activity of theses enzymes induced by exercise. In our study, the mean activity of GPx was increased after a soccer match, however the mean activity of SOD was lower. In both cases, differences post-match did not reach statistical significance. Other conflicting findings have been reported for these enzymes; no increase following exercise was found in the concentration of GPx [34-36], or SOD [35,37,38]. Clearly, these results are likely to depend on the time of sampling, the type of isoenzyme measured (in the case of SOD), the specific sample (plasma, erythrocytes, lymphocytes, neutrophils), the kind of exercise performed, as well as the duration and intensity of exercise, which varies considerably across studies. Furthermore, we found that SOD activity was closely associated with vitamin B6 levels, since players who did not meet with the recommended intake of vitamin B6 showed lower SOD activity immediately post-match. Similar findings were recently reported in rats who when fed with a B6-deficient diet presented lower concentration of SOD activity in kidney [39]. A more pronounced decrease in SOD activity was earlier reported in rats fed a vitamin B6 deficient diet after exercise-induced oxidative stress [40].

Soccer has been described as an aerobic-anaerobic sport in which players’ movements can involve eccentric muscle contractions resulting in muscle fiber and therefore cell breakdown. Some markers, such as CK and LDH, have been used as a way to indicate the grade of cell damage, especially after playing a sport [6,41-44], since microfiber breakdown releases cell content. Thus, serum concentrations of skeletal muscle enzymes constitute a marker of the functional status of muscle tissue and varies widely in both pathological and physiological conditions. Thus, monitoring the concentrations of these markers can help to avoid tissue damage [45]. Although it has long been recognized that there is a close association between dietary carbohydrate intake, muscle glycogen concentration and endurance capacity, these relationships with CK activity are still unknown. In this regard, our study demonstrates that higher carbohydrate intake is associated with a diminished serum concentration of CK and LDH at rest. Several studies have investigated the effect of carbohydrates on the physiological effect induced by exercise, mostly during recovery. For example, after eccentric exercise, no significant effects were found after consuming a higher proportion of carbohydrates [46]. However, muscle recovery cannot be evaluated by changes in serum CK concentrations, as there is no correlation between serum enzyme leakage and muscular performance impairment after exercise [47]. Nevertheless, total creatine kinase levels have been found to depend on age, gender, race, muscle mass, physical activity and climatic condition, and after exercise, CK activity in serum has been found to depend on the level of training [48].

Our study also indicates that nutritional habits may play an important role in CK serum levels as we have found lower concentrations in players who complied with fiber, vitamin B1 and chromium ingestion. The vast majority of published research has centered on carbohydrate intake during exercise and performance [49] or on how a vegetarian diet can affect performance [50,51], but the effect of fiber and other minerals specifically on exercise is still unknown. The current study is the first report that reveals the effects of these nutrients after playing a soccer match. In keeping with the proposals of other authors [52], we hypothesize that increased ingestion of fiber-rich foods, such as wholegrain, fruit and vegetables (which are rich in antioxidant vitamins and minerals), may be a predictor of antioxidant status, and may enhance the protection of tissue cells. This may explain the higher percentage of lymphocytes found post-match in players whose fiber intake was adequate. We also found that adequate vitamin E intake was associated with a less pronounced increase in LDH concentrations after exercise. Vitamin E, as well as vitamin C, is widely known as an important endogenous antioxidant against free radicals [53]. Under conditions of oxidative stress, perhaps these vitamins protect cell membranes and lead to reduced cell breakdown. Similarly, the finding that higher vitamin C intake is related to a higher percentage of lymphocytes immediately post-match corroborates the hypothesis of the protective function of vitamins. Apparently, vitamin C can also stimulate the activity of neutrophils, monocytes and lymphocytes [54] and has been suggested to be implicated in immunoregulation [55]. The antioxidant effect of these vitamins on exercise, however, is controversial. Vitamin E supplementation during exercise does not appear to decrease exercise-induced lipid peroxidation in humans [56]. More recently, another study has demonstrated that vitamin C and E supplementation in soccer players may reduce lipid peroxidation and muscle damage during high intensity efforts [57], but it was not shown to enhance performance [58]. So, our results reinforce the hypothesis of the implication of these vitamins in the stimulation of immune system and the protection of cell breakdown induced by a soccer match.

Little research has been conducted to examine whether exercise increases the need for the B-complex vitamins [59,60]. Some of those vitamins are involved in energy production during exercise and others, such as folic acid and vitamin B12, are required for the production of red blood cells, protein synthesis and tissue repair. In our study, we have also found that adequate folate intake is associated with an improved immune system response after exercise. Folate is frequently low in the diet of female athletes, especially those who have disordered eating patterns [61]. Our results reveal that players who complied with folate intake recommendations exhibited a higher percentage of lymphocytes and a lower percentage of neutrophils just after playing a soccer match. Although deficiencies in B vitamins have not been observed to influence performance, severe deficiency of vitamin B12, folate or both nutrient may result in anemia and in an impaired endurance performance [60,61]. Therefore, it is necessary that athletes consume adequate amounts of these vitamins to support their efforts for optimal performance and a robust immune system.

Broadly speaking, the primary minerals which have been found to be sub-optimal in the diets of athletes, particularly female athletes, are calcium, iron, zinc and magnesium [12], but for many minerals, there are few or even contradictory data about the concentrations found in athletes at rest and during exercise [62]. This is the case of copper and chromium. Copper is a critical mineral involved in many aspects of energy metabolism and an important component for the synthesis of hemoglobin, myoglobin, cytochromes and some peptide hormones [63]. It is also related to the elimination of toxins and free radicals in athletes, as is a cofactor of proteins involved in redox processes. Chromium is involved in a large number of enzymatic processes. It increases tolerance to sugars through the glucose tolerance factor (GTF), a complex of unknown structure, which enhances insulin activity. Clearly, information about these oligoelements is scarce, and so the relevant findings in the present study are of particular interest. Thus, chromium appears to contribute to the prevention of cell damage, since athletes with adequate chromium intake exhibited lower CK activity at rest. Moreover, we found that variations in the percentage of neutrophils and lymphocytes during exercise might be influenced by copper intake, pointing to copper as a non-immune-suppressive mineral.

## Conclusions

The present study contributes to a body of evidence that indicates specific nutrients may influence the antioxidant capacity of soccer players, as well as, cell damage, immunity and the inflammation response induced by playing a match. Thus, the present results concerning nutrition intake should be taken into account by nutritionists and coaches during training sessions and championships, in order to enhance players physiological response to the stress associated with playing a soccer match and eventually, their performance.

## Competing interests

The authors declare that they have no competing interests.

## Authors’ contributions

All authors read and extensively reviewed and contributed to the final manuscript as follows: GL carried out the recollection of the data, designed the dietary booklet and drafted the manuscript. RF carried out the antioxidant analysis. DE participated in the nutrition analysis. LJ participated in the blood analysis measurements and coordination of the study. BA participated in the blood analysis measurements. IJ participated in the design of the study and performed the statistical analysis. GS conceived of the study, and participated in its design and coordination and helped to draft the manuscript. All authors read and approved the final manuscript.
